# Risk factors for hemorrhagic cystitis in children with severe beta-thalassemia after allogeneic hematopoietic stem cell transplantation

**DOI:** 10.3389/fped.2025.1558099

**Published:** 2025-04-03

**Authors:** Hui-Hong Dou, Jian-Ming Luo, Yan-Jun Zhao, Ji-Gan Wang, Yuan-Han Qin

**Affiliations:** ^1^ Department of Pediatrics, Maternal and Child Health Hospital of Guangxi Zhuang Autonomous Region, Guangxi Clinical Research Center for Pediatric Diseases, Nanning, China; ^2^ Department of Pediatrics, The First Affiliated Hospital, Guangxi Medical University, Nanning, China

**Keywords:** hemorrhagic cystitis, HC, beta-thalassemia, allo-HSCT, risk factors

## Abstract

**Purpose:**

To investigate the risk factors for hemorrhagic cystitis (HC) in children with severe beta-thalassemia after allogeneic hematopoietic stem cell transplantation (allo-HSCT).

**Methods:**

The clinical data of 152 children under the age of 15 who underwent allo-HSCT between January 2011 and December 2021 were retrospectively analyzed. The incidence of HC and related variables were evaluated using univariate analysis. Variables with statistical significance (*P* < 0.05) were included in a multivariable logistic regression model to identify independent risk factors for HC.

**Results:**

Among the 152 children, 42 developed HC, with an incidence rate of 27.63%. The median onset time of HC was 25 days (IQR: 10–38.75 days). Univariate analysis indicated that older transplantation age, elevated pre-transplant serum ferritin levels, cytomegalovirus (CMV) infection, and prolonged neutrophil engraftment time were associated with HC occurrence (*P* < 0.05). Multivariable logistic regression further confirmed that older transplantation age (OR 1.236, 95% CI: 1.031–1.531, *P* = 0.033), elevated pre-transplant ferritin levels (OR 1.053, 95% CI: 1.028–1.086, *P* < 0.01), CMV infection (OR 11.522, 95% CI: 2.912–76.345, *P* = 0.002), and prolonged neutrophil engraftment time (OR 1.385, 95% CI: 1.109–1.793, *P* < 0.01) were independent risk factors for HC.

**Conclusion:**

Older transplantation age (>5.95 age years old), elevated pre-transplant serum ferritin levels, CMV infection, and delayed neutrophil engraftment are independent risk factors for HC in children with severe beta-thalassemia after allo-HSCT. Early identification and intervention for these risk factors are crucial in reducing the incidence of HC.

## 1 Introduction

Severe thalassemia (Severe Thalassemia) is one of the most common monogenic hereditary diseases worldwide, with particularly high prevalence in the Mediterranean region, Southeast Asia, and southern China (1, 2). The disease is primarily characterized by defective synthesis of the β-globin chain, leading to chronic anemia, ineffective erythropoiesis, and iron overload, which eventually causes multi-organ damage, including to the heart, liver, and endocrine system (3, 4). For patients with severe thalassemia, traditional treatment primarily relies on regular blood transfusions and iron chelation therapy. However, long-term treatment is costly and associated with transfusion-related complications and a decline in quality of life (5). Allogeneic hematopoietic stem cell transplantation (allo-HSCT) is currently the only potentially curative treatment for this disease and has been widely adopted in clinical practice (6).

Despite the remarkable efficacy of allo-HSCT in treating severe thalassemia, the procedure is complex and associated with various transplantation-related complications, among which hemorrhagic cystitis (HC) is one of the more common and serious complications (7). HC can manifest as aseptic cystitis, microscopic or macroscopic hematuria, and, in severe cases, bladder injury, urinary obstruction, or even transplantation failure (7). Although the mechanisms underlying HC are partially understood—such as bladder damage caused by conditioning regimens, infection with cytomegalovirus (CMV) or BK virus, use of immunosuppressants, and host-related factors—the precise pathophysiology and risk factors remain unclear (8).

In children with severe thalassemia, disease-specific characteristics, such as iron overload caused by long-term transfusions, immune dysfunction, and specific treatment approaches during transplantation (including conditioning regimens and supportive care), may further increase the risk of HC (9). Furthermore, compared to adult patients, pediatric patients may exhibit differences in bladder tolerance and viral infection responses, making research on this particular population especially important. However, studies on the clinical characteristics and risk factors for HC in children with severe thalassemia remain limited.

This study retrospectively analyzed the clinical data of children under the age of 15 with severe thalassemia who underwent allo-HSCT, systematically evaluating the incidence and risk factors of HC. Using univariate and Multivariable logistic regression analyses, the study explored independent risk factors associated with HC occurrence. The findings aim to provide clinical guidance for optimizing peri-transplantation management strategies, reducing complications, and improving transplantation success rates and long-term survival in this patient population.

## 2 Materials and methods

### 2.1 Research design and ethics

This is a retrospective analysis of 152 children under 15 years of age who underwent allogeneic Hematopoietic stem cell thalassemia transplantation between January 2011 to December 2021 at the First Affiliated Hospital of Guangxi Medical University, the study was reviewed and approved by the Ethics Committee of the First Affiliated Hospital of Guangxi Medical University (ref. 2023-E131-01).

### 2.2 Data and variable collection

Patients' clinical records and data were collected by members of the research team, data collected included age, sex, pre-transplant ferritin level, platelet engraftment time, neutrophil engraftment time, presence or absence of Cytomegalovirus (CMV) infection, presence or absence of graft-vs.-host disease (GVHD), Hepatic venous occlusive disease (HVOD), Blood type, Human Leukocyte Antige(HLA)match, presence or absence of fungal infection, with or without sepsis, hypertension, diabetes and other factors.

### 2.3 Preparation before transplantation

#### 2.3.1 High frequency blood transfusion before transplantation

According to the specific situation of the patient, starting 45 days before transplantation, appropriate and frequent blood transfusions should be carried out to maintain hemoglobin levels above 120–140 g/L. The purpose is to provide feedback on the high proliferation of bone marrow through continuous peripheral blood hyperhemoglobinemia, while also improving conditions such as cardiac enlargement, hepatosplenomegaly, and splenic hyperfunction caused by poor blood.

#### 2.3.2 Bone marrow and immune system suppression therapy

Oral hydroxyurea (20–30 mg/kg/day) was given to children one month before transplantation, in order to inhibit and alleviate the hyperproliferation of children's bone marrow, reduce the burden of pretreatment, increase the chances of implantation and reduce rejection after transplantation. Allopurinol tablets were given orally from the beginning of pretreatment to the day before transplantation to prevent renal tubular injury caused by chemotherapy drugs.

#### 2.3.3 Pretreatment scheme

The pretreatment scheme using busulfan (BU) + cyclophosphamide (CY) + fludarabine (FLU) + antithymocyte globulin (rabbit) (ATG). The specific usage is as follows:

Busulfan (Busulfan, BU):dose16 mg/kg. It is administered intravenously for four days. (−9d⁓−6d)
Fludarabine (FLU): dose 150 mg/M2. It is administered intravenously for three days. (−11d⁓−9d)
Cyclophosphate (CY): dose 200 mg/kg. It is administered intravenously for four days. (−5d⁓−2d)

Antihuman thymus immunoglobulin (ATG): dose 6–10 mg/kg. Divide it into four intravenous drip.

#### 2.3.4 Definition of hemorrhagic cystitis (HC)

After the transplantation, the children appear to be in a microscopic or macroscopic hematuria, with or without urinary frequency, urgency, urinary pain and other bladder irritation symptoms. Cystoscopy showed local or diffuse bleeding and inflammatory changes in the bladder mucosa. Exclusion of bacterial infection, drug-induced hematuria and other, such as disseminated intravascular coagulation, multiple organ dysfunction or sepsis, It can be diagnosed. According to the degree of hematuria, the clinical classification of HC is as follows (10):

Ⅰ degrees: Hematuria under microscope
Ⅱ degrees: Gross hematuria
Ⅲ degrees: Naked eye hematuria with clot
Ⅳ degrees: Clot obstruction urethra, this should take measures to remove blood clots or require surgical intervention.

Neutrophil implantation: peripheral blood neutrophil count >0.5 × 10 9/L for 3 days; Platelet implantation: peripheral platelet count >20 × 10 9/L for 7 days and no platelet transfusion.

### 2.4 Statistical methods

Statistical analysis was performed using R4.3.1 statistical software. The measurement data were tested by Shapiro–Wilk test for normality, and the normal distribution data were expressed as mean ± standard deviation. The measurement data among different groups were analyzed by *T*-test or Analysis of Variance (ANOVA). The non-normal distribution data were expressed as the median and interquartile interval M (P25, P75), and were compared between different groups using the Mann–Whitney U rank sum test or Kruskal–Wallis H test. The count data were expressed by frequency, and *X^2^* test was used for comparison between groups. Some patients also had missing values for other variables. We used multiple imputation by chained equations (MICE) to fill in these missing values, which included GVHD, diabetes, sepsis, fungal infections, and others. The risk factors were analyzed by binary logistic regression. *P* < 0.05 indicated that the difference was statistically significant.

## 3 Results

### 3.1 General characteristics of patients

The clinical characteristics of 152 children under the age of 15 with severe beta-thalassemia are summarized in Table 1. The median age was 4 years (IQR: 3–6 years), with 108 male and 44 female patients. Among the 152 children, 42 developed hemorrhagic cystitis (HC) after transplantation, with an incidence rate of 27.63%. The median time of HC onset was 25 days (IQR: 10–38.75 days). Based on severity, 16 cases (38.10%) were classified as mild HC (grades I–II), while 26 cases (61.90%) were classified as severe HC (grade III and above). Among the HC cases, 1 early-onset HC case resulted in death, whereas the other 41 cases were late-onset and survived.

**Table 1:** Clinical characteristics of hemorrhagic cystitis after transplantation in thalassemia patients.

Patient characteristics	All patients	NO-Hemorrhagic cystitis	Hemorrhagic cystitis	*P value*
Occurrence_time(IQR), days	25 (10–38.75)	NA	25 (10–38.75)	0.95
CD34(IQR), 10^6^/kg	7.92 (2.43–20.2)	6.8 (2.14–17.88)	14.27 (3.55–21.83)	0.2
Ferritin(IQR), ng/ml	6,686 (4,467–9,719.89)	5,073.5 (3,293.82–6,959.5)	10,377 (8,020–14,129.12)	<0.01
MNC(IQR), 10^8^/kg	10.38 (1.8–20.79)	7.78 (1.66–18.32)	17.59 (1.91–22.22)	0.1
Live_MNC(IQR), 10^8^/kg	5.42 (1.04–17.32)	3.77 (0.99–16.54)	8.05 (1.27–18.73)	0.35
Neutrophil_implantation_time (IQR), days	11 (10–13)	11 (10–12)	13 (11–15)	<0.01
PLT_day (IQR), days	13.5 (11.25–15.75)	13 (12–16)	14 (11–15)	0.58
Age(IQR), Years	4 (3–6)	3.8 (3–5)	5.95 (4.25–8)	<0.01
Sex, Female, *n*(%)	44 (28.95)	31 (28.18)	13 (30.95)	0.89
Sex, Male, *n* (%)	108 (71.05)	79 (71.82)	29 (69.05)	
ɑ and β thalassemia, *n*(%)	8 (6.50)	4 (4.49)	4 (11.76)	0.29
β-thalassemia, *n*(%)	115 (93.50)	85 (95.51)	30 (88.24)	
HLA Full Match, *n*(%)	146 (96.05)	106 (96.36)	40 (95.24)	0.91
HLA Partial Match, *n*(%)	6 (3.95)	4 (3.64)	2 (4.76)	
Blood_type, A+, *n*(%)	36 (25.17)	27 (25.47)	9 (24.32)	0.44
Blood_type, AB+, *n*(%)	7 (4.90)	4 (3.77)	3 (8.11)	
Blood_type, B+, *n*(%)	32 (22.38)	26 (24.53)	6 (16.22)	
Blood_type, O+, *n* (%)	68 (47.55)	49 (46.23)	19 (51.35)	
Donor_blood_type, A+, *n*(%)	26 (19.40)	18 (18.37)	8 (22.22)	0.94
Donor_blood_type, AB+, *n*(%)	9 (6.72)	6 (6.12)	3 (8.33)	
Donor_blood_type, B+, *n*(%)	36 (26.87)	26 (26.53)	10 (27.78)	
Donor_blood_type, O+, *n*(%)	63 (47.01)	48 (48.98)	15 (41.67)	
Donor_gender, Female, *n*(%)	72 (52.94)	50 (51.02)	22 (57.89)	0.66
Donor_gender, Male, *n*(%)	64 (47.06)	48 (48.98)	16 (42.11)	
Blood Type Compatibility, NO, *n*(%)	55 (38.46)	39 (37.50)	16 (41.03)	0.9
Blood Type Compatibility, YES, *n*(%)	88 (61.54)	65 (62.50)	23 (58.97)	
Gender_consistency, NO, *n*(%)	81 (58.27)	56 (55.45)	25 (65.79)	0.36
Gender_consistency, YES, *n*(%)	58 (41.73)	45 (44.55)	13 (34.21)	
CMV-IgM, Negative, *n*(%)	54 (40.91)	51 (54.84)	3 (7.69)	<0.01
CMV-IgM, Positive, *n*(%)	78 (59.09)	42 (45.16)	36 (92.31)	
HVOD, NO, *n*(%)	137 (90.73)	102 (92.73)	35 (85.37)	0.28
HVOD, YES, *n*(%)	14 (9.27)	8 (7.27)	6 (14.63)	
Diabetes, NO, *n*(%)	146 (96.69)	106 (96.36)	40 (97.56)	0.95
Diabetes, YES, *n*(%)	5 (3.31)	4 (3.64)	1 (2.44)	
Hypertension, NO, *n*(%)	104 (68.42)	74 (67.27)	30 (71.43)	0.77
Hypertension, YES, *n*(%)	48 (31.58)	36 (32.73)	12 (28.57)	
Sepsis, NO, *n*(%)	125 (82.78)	90 (81.82)	35 (85.37)	0.74
Sepsis, YES, *n*(%)	26 (17.22)	20 (18.18)	6 (14.63)	
Pneumonia, NO, *n*(%)	87 (57.24)	60 (54.55)	27 (64.29)	0.37
Pneumonia, YES, *n*(%)	65 (42.76)	50 (45.45)	15 (35.71)	
Fungus, NO, *n*(%)	140 (93.33)	103 (93.64)	37 (92.50)	0.99
Fungus, YES, *n*(%)	10 (6.67)	7 (6.36)	3 (7.50)	
GVHD, NO, *n*(%)	110 (72.85)	84 (76.36)	26 (63.41)	0.19
GVHD, YES, *n*(%)	41 (27.15)	26 (23.64)	15 (36.59)	

IQR, interquartile range; GVHD, graft versus-host disease; HVOD, hepatic veno-occlusive disease.

Univariate analysis identified that older age at transplantation (>5.95 age years old), elevated pre-transplant ferritin levels, cytomegalovirus (CMV) infection, and prolonged neutrophil engraftment time were high-risk factors for HC in children with severe beta-thalassemia undergoing allo-HSCT.

### 3.2 Multivariable logistic regression analysis of HC risk factors

A Multivariable analysis using unconditional logistic regression was performed on the four variables identified as statistically significant in univariate analysis. The results confirmed that these four variables remained statistically significant:

• Older age at transplantation (OR 1.236, 95% CI: 1.031–1.531, *P* = 0.033)
• Elevated pre-transplant ferritin levels (OR 1.053, 95% CI: 1.028–1.086, *P* < 0.01)
• CMV-IgM positivity (OR 11.522, 95% CI: 2.912–76.345, *P* = 0.002)
• Prolonged neutrophil engraftment time (OR 1.385, 95% CI: 1.109–1.793, *P* < 0.01)

The logistic regression analysis results are detailed in Table 2.

**Table 2:** Logistic regression analysis results of HC risk factors.

VariableName	Estimate	SE	*Z*_value	*P* value	OR	95% (CI)
(Intercept)	−10.0671	1.8367	−5.4810	0.0000	0.0000	0.0000–0.221
CMV-IgM	2.444	0.799	3.057	0.002	11.522	2.912–76.345
Ferritin	0.051	0.014	3.739	<0.01	1.053	1.028–1.086
Age	0.213	0.099	2.137	0.033	1.238	1.031–1.531
Neutrophil_implantation_time	0.326	0.122	2.667	<0.01	1.385	1.109–1.793

## 4 Discussion

HC is mainly the result of diffuse inflammation and vascular damage of the bladder mucosa caused by immune suppression and opportunistic infections. The pathological basis of HC is that various factors directly or indirectly cause damage to the bladder mucosa, promoting congestion and edema, forming ulcers, and subsequently bleeding and necrosis. Early onset HC is related to factors such as the use of high-dose alkylating agents in Conditioning regimens, radiation damage, and thrombocytopenia; The pathogenesis of delayed onset HC is relatively complex, and the influencing factors are diverse (11). Clinical manifestations include signs and symptoms related to cystitis, such as age stimulation, difficulty urinating, urgency and heaviness, lower abdominal pain associated with hematuria, and urinary tract thrombosis (12).

High-dose ATG (10 mg/kg) is now recognized as an independent risk factor for the development of HC, and ATG is a potent immunosuppressive agent that prevents GVHD and is increasingly used in haploidentical transplant patients. Many studies have demonstrated that T-cell function recovery is significantly delayed in patients treated with ATG, which significantly affects the reconstitution of immune function, and that high-dose ATG has a more substantial inhibitory effect on immune reconstitution; It can increase the risk of infection, promote the reactivation of latent infection such as BKV and CMV, and induce the incidence of HC (13). Toxic metabolites of cyclophosphamide (acrolein) induce a complex inflammatory response that impairs the integrity of the urinary tract epithelium, with swelling of the bladder mucosa, bleeding, and ulcers (14). Because ATG, busulfan and cyclophosphamide were used in all the pretreatments, no comparative analysis was made.

Cytomegalovirus infection is one of the high-risk factors for hemorrhagic cystitis after transplantation. Studies have shown that CMV infection plays an important role in inducing HC, as HSCT transplant recipients receive high-dose radiation/conditioning reginem in the early stage, leading to significant immune dysfunction, virus invasion or latent virus activation in the body, damaging the bladder mucosa, which is consistent with the conclusion of Zhang L (15).Some researchers also believe that CMV and BKV infection co-cause HC, and CMV infection may promote the amplification of BKV, thus leading to the occurrence of HC (16). Due to limited conditions, BK virus was not detected in patients after transplantation in the early stage of this study, so BK virus was not included in the analysis.

In this study, it was found that the delayed engraftment time of neutrophils was one of the risk factors. Some researchers (17) found in the mouse model that neutrophils played a certain role in cyclophosphamide induced HC, suggesting that granulocyte colony stimulating factor (G-CSF) could promote the migration and aggregation of neutrophils to the damaged bladder mucosa and induce the occurrence of inflammation. In patients with slow neutrophil implantation after allo-HSCT, this may be facilitated by prolonged use of G-CSF to promote hematopoiesis. The study also found that without mucosal damage, G-CSF alone did not induce neutrophil migration and tissue damage, it also confirmed that mucosal damage during conditioning reginem may be a prerequisite for Hemorrhagic cystitis.

We found that patient age was a risk factor for HC, but in adult studies, age was not a risk factor (18). Regarding age as a risk factor, it is possible that young children may not have developed a habit of urination and frequent urination will excrete CTX (19). Ferritin is also a risk factor, and pre-transplantation ferritin levels are associated with infection. High ferritin levels may lead to more infection rates, and organ damage induced by large iron deposits may involve the liver, pancreas, endocrine system and heart (20). Iron overload can lead to injury of hematopoietic function and decrease of hematopoietic ability, thus affecting proliferation and apoptosis of hematopoietic microenvironment, and decreased hematopoietic support ability (21), resulting in delayed engraftmen of donor grafts and easy co-infection. Iron overload leads to decreased immune function of the body and virus entry, which can also be explained. Iron overload leads to the enhancement of lipid peroxidation and the decrease of antioxidant enzymes, which leads to the enhancement of oxidative stress and the renal tubule interstitium and the transitional epithelium of the bladder, etc., which may aggravate the damage to the urinary system and lead to the occurrence of HC (22).

At present, BK virus is recognized as an independent risk factor for HC, and BKV was detected in the urine of 80% of HC patients after HSCT. BK virus reactivation is one of the main causes of HC after HSCT. The virus hides in renal tubules and urothelial cells. During the immunosuppressor phase, BKV reactivation occurs, and its uncontrolled replication in urothelial cells may further lead to denudation of the damaged bladder mucosa (23). Due to the limited conditions in our hospital, there was no BK virus related detection in the early stage, and the failure to include BK virus in the analysis in this paper is a major defect of this study.

The incidence of hemorrhagic cystitis (HC) in children with severe β-thalassemia undergoing allogeneic hematopoietic stem cell transplantation (allo-HSCT) appears to be lower than that in patients with other malignant diseases. In this study, 27.5% of patients developed HC, whereas a study involving 153 adult patients who underwent haploidentical large-dose peripheral blood stem cell transplantation for leukemia found that 64 patients developed HC, with an incidence of 41.8% (24). Additionally, another study from China reported that among 89 pediatric and adolescent patients (including 44 cases of acute lymphoblastic leukemia (ALL), 33 cases of acute myeloid leukemia (AML), 3 cases of acute hybrid leukemia (AHL), and 9 cases of myelodysplastic syndrome (MDS)) who underwent haplo-HSCT, 32 cases (36%) developed HC (25). The specific reason for this difference may be attributed to the fact that the current study is a single-center study with a relatively small sample size, and thus, the findings may not be conclusive.

In conclusion, HC is an common complication after allogeneic hematopoietic stem cell transplantation, and clinical data from our center show that its occurrence is related to age, prolonged neutrophil implantation time, CMV infection, and elevated ferritin. Therefore, iron removal before transplantation, recovery of blood image as soon as possible after transplantation, promotion of immune reconstruction, and preemptive treatment of CMV virus may be the key to reduce LOHC occurrence and improve the quality of life of patients.

## Data Availability

The original contributions presented in the study are included in the article/Supplementary Material, further inquiries can be directed to the corresponding authors.
